# Chicago Medical Society

**Published:** 1886-06

**Authors:** 


CHICAGO MEDICAL SOCIETY.

*Stated Meeting April 19, 1886.* THE PRESIDENT, E. J. DOERING, M. D., IN THE CHAIR.

PROFESSOR G. C. PAOLI read a paper entitled

THE REASONS WHY FEMALE PHYSICIANS ARE DESIRABLE IN INSANE ASYLUMS.

He demonstrated that the most eminent specialists in psychiatry in the United States are a unit in maintaining that female physicians are best qualified for treating the female insane. Many states have passed laws requiring one female physician to be allotted to each one hundred female insane in their asylums.

DR. J. C. KIERNAN said eminent authorities had already pointed the relation which exists between uterine disease and insanity. Popular opinion is growing in favor of the employment of female physicians in the treatment of the female insane.

DR. WM. T. BELFIELD reported nine cases of

IMPERMEABLE STRICTURE TREATED BY ELECTROLYSIS.

During the past two years he has treated thirty-seven cases of stricture by electrolysis; and except for strictures located within an inch of the meatus, and for strictures of large calibre elsewhere, considers it preferable to dilatation and urethrotomy for the following reasons: 1. It will pass through any stricture, however tight, rigid, long or tortuous. 2. As a rule it causes no pain, bleeding, chill, nor urethral fever. 3. It is always devoid of danger. 4. Its effects are lasting.

In certain numerous cases electrolysis is not merely a preferable, but really the only practicable treatment. Such are, 1, old, rigid, cartilaginous strictures in men of middle or advanced age, where urethrotomy is dangerous and dilatation ineffectual; 2, impermeable strictures; 3, tight and rigid strictures with perineal or scrotal fistulæ.

DR. BELFIELD then narrated the successful treatment by electrolysis of nine such cases. In three of these there was complete retention of urine, the bladder being distended above the umbilicus; these strictures were absolutely impermeable to urine from within as well as to instruments from without. In each case a No. 10 electrode (French) was passed into the bladder in less than twenty minutes, permitting the immediate introduction of a catheter. In each of these cases perineal section would have been, without the battery, inevitable.

In the remaining six cases Dr. Belfield, as well as other surgeons, had failed in attempts to pass bougies into the bladder; yet as the patients were still enabled to force a feeble, dribbling stream outward, these strictures were theoretically permeable, though practically impermeable. In these also a small electrode readily entered the bladder in one or two sittings.

In one case, seen in consultation with Dr. Miller, the patient had a series of tight, rigid, impermeable strictures, and twenty-seven fistulous openings in the scrotum and perineum ; he had submitted to both internal and external urethrotomy, and to numerous unsuccessful attempts at dilatation; was urinating every half hour, day and night. In fifteen minutes a No. 10 bulb entered the bladder ; that night patient rose only once to urinate, and for the first time in several years the urine flowed entirely from the meatus and not from the fistulæ.

The unfavorable results obtained by various physicians in their attempts at electrolysis have been caused by the use of improper currents, whereby heat was generated and the urethra cauterized, causing violent inflammation and even extensive sloughing. When properly used, the heat produced is insignificant; with six to fifteen small cells and a weak fluid, the cicatricial tissue constituting the stricture is dissolved away but not cauterized. Since cicatricial tissue is but scantily supplied with blood, and is therefore poorly nourished, it yields to a dissolving current which is insufficient to disturb the healthy urethral tissues.

DR. L. L. MCARTHUR said he had treated with success by electrolysis one case in which numerous operations had already been performed. When patient came to him he could pass a No. 8 sound; used No. 9 electrode, and the patient can now pass No. 15 American sound. Never had any pain or difficulty in micturating since the operation.

DR. M. B. BROWN detailed two cases of impermeable and two of permeable stricture, in which he used electrolysis with good results.

PROFESSOR C. FENGER said he must confess that he was somewhat surprised by seeing the announcement of Dr. Belfield's paper " Nine Impermeable Strictures Treated by Electrolysis." He had tried electrolysis a few years ago, but never had any success from it. It surprised him that Dr. Belfield should meet with nine impermeable strictures in a very short period of time, while Sir Henry Thompson has only met with three impermeable strictures in his whole life. By hearing the paper read, however, he understood that Dr. Belfield does not mean to say that the strictures were impermeable in the strict sense of the word—the only correct one—but only meant that it was difficult to pass any instrument through these strictures. Sir Henry Thompson, in his "Diseases of the Urinary Organs," of 1882, p. 28, says: "Impermeability cannot be held to describe a character, a physical quality of the stricture itself, but rather indicates the quality of the surgeon who has treated it." Impermeable stricture is a contradiction in terms; it is not heard of so much now as it was twenty years ago. Dittl, of Vienna, in speaking about relative and absolute impermeable strictures, says a stricture may be impermeable for one man, or at a certain time, and permeable for another man, or at another time. Other modern authors on surgery, as König and Albert, speak about the matter in a similar way.

The second surprise for him was the advocated treatment by electricity, or electrolysis. There has always been in his mind a suspicious halo of mysticism about the electrolysis, whether applied to the different forms of surgical tumors or to strictures of the uretha. He understands from the paper that the electrolysis does not mean galvano-caustic treatment, although quite recently Jardin, of Paris, uses a small galvano-cautery knife for passing slowly through the stricture. Dr. Belfield warns very justly against cauterization. The non-caustic electrolysis is to me a very mystic process. Professor Fenger remembered years ago one of Billroth's clinics in which he spoke about electrolytic treatment of venous angiomas of the face, that he expressed as the result of his experience the following: "The electrolytic needle has no more or other effect on the tumor in question than the mere mechanical disturbance of the tissue elements; that is, than any other needle not connected with a battery would produce."

Frankly, Professor Fenger said that the historical fate of electrolysis in strictures as well as elsewhere, up to date, has invariably been the following: Ever since Tripier, in 1864, and Mallez, in 1872, applied the electrolysis in strictures of the urethra, this method of treatment has come to the surface once every two or three years only to disappear again, and it has never been able to take any hold on the profession; not because it has not been tried, but rather because it has not been found superior, or even equal, to the other methods. Littel states that, on the rather promising representations of Tripier and Mallez, he tried electrolysis in three cases of very narrow stricture. It proved of no effect in any of the cases, and in one of them a local inflammation followed. Sir Henry Thompson does not even mention the electric treatment anywhere in his writings about strictures, but warns very emphatically against any method of cauterization. König says, in a very short appended notice, that only the short and soft strictures depending upon a polypus or warty growth of granulation tissue are proper objects for cauterization, either chemical (Duchamp) or galvano-caustic (Middeldorf). Otis, our American authority in this line of surgery, does not mention electrolysis with a word.

Newman, of New York (*Medical Record*, August 12-19, 1882), is not only the advocate of electrolysis in this country, but has written so assiduously and specified the method so minutely as to have it termed, in the foreign literature, "Newman's method." In 1882 Newman's old and new cases numbered twenty-three only. In reporting Newman's articles for Virchow's *Jahresbericht*, Güterbock, of Berlin, says that "Newman's method has already, in 1872 and 1876, been criticised so thoroughly that not much more need be said about it." Dikmann, in New York *Medical Record*, Jan. 5, 1884, reports twenty-eight cases. Gräf, in Norway, reports in 1884, two cases treated by "Newman's method," and Verneuil, in 1884, recommends Jardin's method—that is, cauterization.

Thus the electrolytic treatment of stricture has been tried off and on for over twenty years, but has only taken hold here and there, sporadically, and for a short time. It has in the urethral, not any more than in the other fields of surgery, as yet to any extent replaced the more rational treatment by mechanical means.

The electrolysis may, however, have a further trial, and if the success in extensive resilient strictures, as in one of Dr. Belfield's cases, should prove to hold good for other cases of that kind, it is possible that this treatment will have a better fate in the future than it has had in the past.

DR. R. TILLEY said he did not consider electrolysis to be the proper term to be used in connection with the use of electricity in the manner suggested. Electrolysis, in the ordinary acceptation of the term, means the decomposition by electricity of water, or tissues, being in the nature of a chemical decomposition. He would like to know how far apart the electrodes were placed. (Dr. Belfield replied that the negative electrode was placed in the urethra, and the positive pole in any position on or near the penis, according to the effect intended). He could not see how electrolysis could be produced with the electrodes so far apart, and a weak fluid, without a cauterizing effect. From his extensive experience of cocaine in producing contraction of the tissues in the nose, he would expect to secure as good if not better results, reasoning by analogy, in its use in strictures of the urethra as in the use of electricity. He would think its internal application would be followed by wonderful results.

DR. W. T. BELFIELD closed the discussion by saying, in reply to Dr. Fenger's criticism, that these strictures were not impermeable, he would remind him that he called six of them "theoretically permeable, practically impermeable;" that is, a feeble stream was forced through, but no instrument could be introduced. The remaining three were absolutely impermeable to urine as well as instruments. They were water-tight, the bladder in each case being immensely distended. Dr. Fenger, in the cases where he attempted electrolysis, evidently made the usual mistake: he employed a strong current and *cauterized*, but did not electrolyze, the stricture. With a proper current he would probably have a better result. Sir Henry Thompson and Professor Dittl do not, it is true, endorse electrolysis; but he knows that neither of them has ever fairly tried it; they have contented themselves with the vague, unfavorable verdict of those who, having once or twice misused the current, attribute to the battery the faults which belong to themselves. Thompson's conservatism and intolerance are notorious; he bitterly opposed Bigelow's litholapaxy (without trying it) until it was adopted by everybody else.

Possibly, as Professor Fenger says, there is no "urgent necessity" for any other method than urethrotomy and dilatation; but surgeons who have to treat many such strictures as those described in the paper under discussion, think otherwise. These methods certainly possess one point of national economic value which electrolysis lacks, namely: they assist in repressing the excess of population by quietly removing many patients.

Electrolysis "bobs up" and down only when ignorantly used; when properly employed it comes to stay.

To Dr. Tilley he would say that the action is properly termed "electrolysis;" it is the same effect as is seen in the decomposition of water, and is produced by the same current. During the action of the current a white foam and bubbles come up alongside the instrument. Cocaine causes contraction of blood-vessels in the urethra as well as in the nose; but it cannot, so far as he is aware, remove cicatricial tissue in either locality.

In conclusion, he would repeat that the secret of success lies in securing chemical force and avoiding heat; the former removes the stricture easily and painlessly; the latter causes violent inflammation and sloughing.

OSTEO-PLASTIC RESECTION.

PROFESSOR C. FENGER then presented a patient before the Society with the following words: I wish to bring this patient before the Society to illustrate the results of an operation called "osteo-plastic resection of the foot," and devised by Wladimnoff and Mikulicz. The operation consists in removal of the heel, soft parts and bones, and then uniting the remainder of the foot to the tibia in the position of an artificial pes equinus. As you will see, the patient has on a plaster cast from the toe to below the knee, and is able to walk in this cast without crutch or cane. He limps because the leg operated upon is two inches longer than the other one, and not because of inability to step on the leg. He will have in future to wear a high sole or heel under the well foot. While this plaster cast is being taken off I shall pass round the specimen of the removed heel and say a few words about the operation.

The patient had suffered for one year from a chronic traumatic osteomyelitis of the tarsus resulting in anchylosis of the joints, fistulæ on dorsal side of tarsus and a loss of substance of the skin of the heel. Pirogoff's or Syme's operation being out of the question, the choice was only between a supramalleolar amputation and the osteo-plastic resection. This operation was performed fourteen months ago. Union of the bones took place in from four to six months, and it is only the subsequent small operations for bringing the toes in dorsal flexion that have required so long a time before the patient has been able to commence to walk.

The plaster cast being now removed, you will find the foot in the axis of the leg in equinus position, the toes dorsally flexed to a right angle with the foot. There is active flexibility of the toes and some active mobility of the foot. This mobility, however, does not take place between the united surfaces of the tibia and fibula on the one side, and the scaphoid and cuboid bones on the other; as can be seen by examining the prominences representing the rudimentary newly formed malleoli—but the mobility takes place in the joints of the metatarsus. As to the question whether these joints will be able to bear the strain of the weight of the body during walking with the foot in this abnormal position, this patient of course proves nothing as yet. But from the other cases operated upon, in all nineteen, we can conclude that we have the right to expect a useful foot for walking. A patient operated upon by Socin, in Basle, is able without boot—the boot devised by Miculicz, which I now pass round for inspection—or cane to walk, but can walk all day long and perform a farmer's work in the field. You will further notice that the walking surface of the foot, being the plantar surface of the heads of the metatarsal bones and the toes, is considerably larger than the surface which either a Syme's or a Pirogoff's operation would leave the patient to walk on. This, together with the active mobility of the toes, is regarded as an advantage that functionally places the osteo-plastic resection superior to the two other operations mentioned.

We find by examining the foot everywhere painless on pressure, and consequently there is nowhere any recurrence of the disease.

Miculicz feared that the anterior tibial artery might not be sufficient for the blood-supply of the foot, his method of operating dividing all the branches of the posterior tibial artery. I had the same apprehension, and changed the incision so as to save one of the terminal branches of the last-named vessel, namely, the internal plantar artery. This fear is not unfounded, as in one of Sordina's cases operated upon according to Miculicz's description, gangrene of the foot necessitated supramalleolar amputation on the fourth day. The mortality of the operation is as yet none, but as all the nineteen cases have fallen within the last few years except Wladimnoff's, and consequently have been treated antiseptically, it cannot be said that the danger is less than either in Syme's or Pirogoff's operation. There is no doubt that the functional results are far superior to that of a supramalleolar amputation even if the patient will always have to wear the Miculicz boot. Consequently it may be safe to say that osteo-plastic resection has already a legitimate place, although perhaps as yet not strictly enough defined, in the surgery of the tarsus.

The Society adjourned.

